# Comparison of the variability in mortality data generated by CDC bottle bioassay, WHO tube test, and topical application bioassay using *Aedes aegypti* mosquitoes

**DOI:** 10.1186/s13071-022-05583-2

**Published:** 2022-12-20

**Authors:** Rachel A. Althoff, Silvie Huijben

**Affiliations:** 1 The Center for Evolution & Medicine, School of Life Sciences, Arizona State University, Tempe, AZ USA; 2 Computational and Modeling Sciences Center, Simon A. Levin Mathematical, Arizona State University, Tempe, AZ USA

**Keywords:** Insecticide resistance, Susceptibility bioassay, CDC bottle bioassay, Topical application bioassay, WHO tube test, Public health policy, Surveillance

## Abstract

**Background:**

Insecticide resistance remains a major public health problem. Resistance surveillance is critical for effective vector control and resistance management planning. Commonly used insecticide susceptibility bioassays for mosquitoes are the CDC bottle bioassay and the WHO tube test. Less commonly used in the field but considered the gold standard for assessing insecticide susceptibility in the development of novel insecticides is the topical application bioassay. Each of these bioassays has critical differences in how they assess insecticide susceptibility that impacts their ability to differentiate between resistant and susceptible populations or determine different levels of resistance intensity.

**Methods:**

We compared the CDC bottle bioassay, the WHO tube test, and the topical application bioassay in establishing the dose–response against deltamethrin (DM) using the DM-resistant *Aedes aegypti* strain MC1. Mosquitoes were exposed to a range of insecticide concentrations to establish a dose–response curve and assess variation around model predictions. In addition, 10 replicates of 20–25 mosquitoes were exposed to a fixed dose with intermediate mortality to assess the degree of variation in mortality.

**Results:**

The topical application bioassay exhibited the lowest amount of variation in the dose–response data, followed by the WHO tube test. The CDC bottle bioassay had the highest level of variation. In the fixed-dose experiment, a higher variance was similarly found for the CDC bottle bioassay compared with the WHO tube test and topical application bioassay.

**Conclusion:**

These data suggest that the CDC bottle bioassay has the lowest power and the topical application bioassay the highest power to differentiate between resistant and susceptible populations and assess changes over time and between populations. This observation has significant implications for the interpretation of surveillance results from different assays. Ultimately, it will be important to discuss optimal insecticide resistance surveillance tools in terms of the surveillance objective, practicality in the field, and accuracy of the tool to reach that objective.

**Graphical Abstract:**

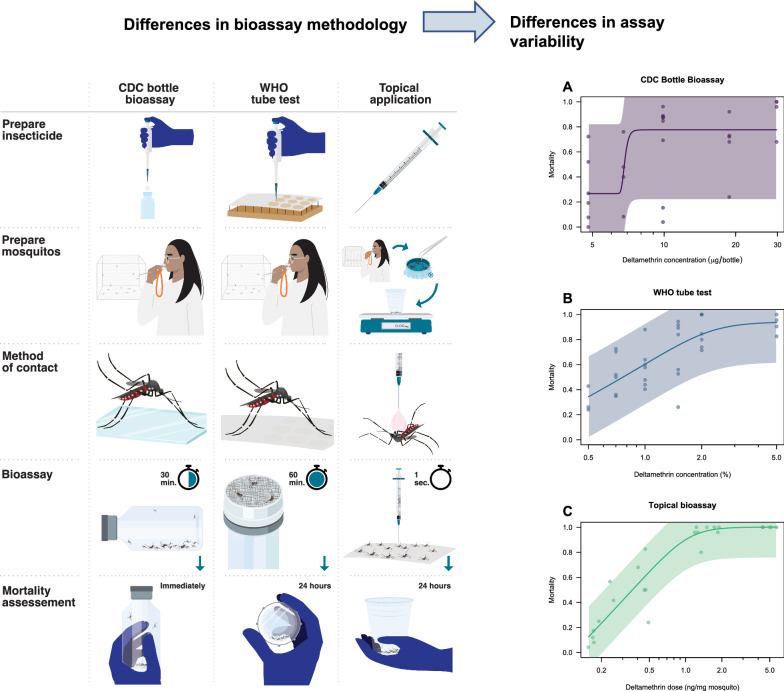

**Supplementary Information:**

The online version contains supplementary material available at 10.1186/s13071-022-05583-2.

## Background

Insecticide resistance is a continuing evolutionary problem. It has contributed to the perpetuation of mosquito-borne diseases, including Zika, dengue, chikungunya, and malaria, due to the failure of vector control interventions such as fogging and insecticidal bednets [1–4]. Intense insecticide resistance surveillance of old and new insecticides, and improved resistance management strategies are crucial to effectively control vector-borne diseases [2, 5, 6]. Surveillance data are used to identify resistance profiles in a given area, which can help identify when and where resistance is emerging or spreading and inform resistance prevention strategies. Early detection of resistance allows for a change of insecticide class use, and accurate data collection in these settings is imperative, as it is important to not under- or overestimate resistance. Underestimation could lead to the continued use of a failing chemical and hence reduced vector control, the waste of resources (money and time) by applying an inefficacious product, and the potential selection of higher levels of resistance in the mosquito population. Such false-negative observations could occur due to insufficient sensitivity of the assay to accurately detect mortality lower than 90 or 97%, small sample size used (leading to spurious results because of random variation), inaccuracy in assay preparation (mosquitoes pick up more insecticide than intended), rough handling (mosquitoes are killed by rough handling instead of insecticide exposure), or inaccurate mortality assessment. On the other hand, overestimation could lead to effective insecticides unnecessarily being replaced with novel ones, which could be particularly problematic when these alternative insecticides are more expensive and thus reduce the coverage of households that can be treated. Such false positives could similarly occur due to a lack of sensitivity of the assay to accurately detect mortality lower than 90 or 97%, too small sample sizes, inaccuracy of preparation of the assay (exposing mosquitoes to too small dosages), and inaccuracy of assessing mortality (for instance, identifying mosquitoes with erratic flight as alive, whereas it follows the definition for “dead” [5, 7]). Therefore, it is important to keep the bioassay preparation standardized, mortality assessment as objective as possible, and choose assays with the highest level of accuracy [8]. Since obtaining sufficient mosquito numbers is a problem in many areas, a bioassay with a low level of variation is preferable as this reduces the number of mosquitoes needed to be confident in the classification of a resistant versus susceptible population. However, the level of inherent variation that each bioassay produces has never been fully established.

The most common bioassays for routine surveillance of adult malaria vectors are threshold assays, where the phenotypic response of a vector sample is measured after exposure to a diagnostic concentration of an insecticide (typically twice the lethal concentration killing 99% of susceptible mosquitoes). If mortality is between 90 and 97%, populations are defined as having “suspected resistance,” and the assay should be repeated to confirm resistance. If mortality falls below 90%, the population is defined as confirmed resistant [5]. Following a first threshold assay, intensity assays could be performed to determine survival at higher dosages, typically 5× and 10× the diagnostic concentration, to determine whether the resistance intensity is low, moderate, or high [5]. Such different levels of resistance intensity have been associated with the degree to which vector control tools such as long-lasting insecticidal nets (LLINs) are effective following multiple exposures [9]. However, in general, these assays are designed to establish the technical resistance of a population: to assess whether the population has changed their phenotypic response over time or space. The predictive value of such assays regarding the effectiveness of a vector control tool in the field is limited [5, 10]. The two most commonly used threshold bioassays are the World Health Organization (WHO) tube test [5] and the Centers for Disease Control and Prevention (CDC) bottle bioassay [7], both of which expose mosquitoes to insecticides via tarsal contact (i.e., mosquito lands on a treated surface). The WHO tube test involves introducing mosquitoes into a plastic tube lined with paper coated with insecticide and a carrier oil. The CDC bottle bioassay involves aspirating mosquitoes into a glass bottle coated with an insecticide. Recently, the WHO bottle bioassay was introduced as a modified version of the CDC bottle bioassay for testing active ingredients such as pyriproxyfen that, due to their chemical properties, prevent their impregnation on filter papers [5, 11]. Besides threshold assays, there are bioassays that establish dose–response curves that allow for the calculation of resistance ratios relative to a susceptible strain, such as the topical application bioassay. Mosquitoes are dosed individually with a range of insecticide doses [12–14]. The topical application bioassay is considered the gold standard for toxicology studies and is recommended by WHO in phase I studies to determine the toxicity of insecticides and assess cross-resistance [15]. While the topical application bioassay is occasionally used as an insecticide resistance surveillance tool (e.g., [16–19]) and was recently recommended as a new surveillance tool for orally ingested insecticides [14], it is not routinely used in malaria-endemic areas to monitor resistance in malaria vectors.

**Fig. 1:**
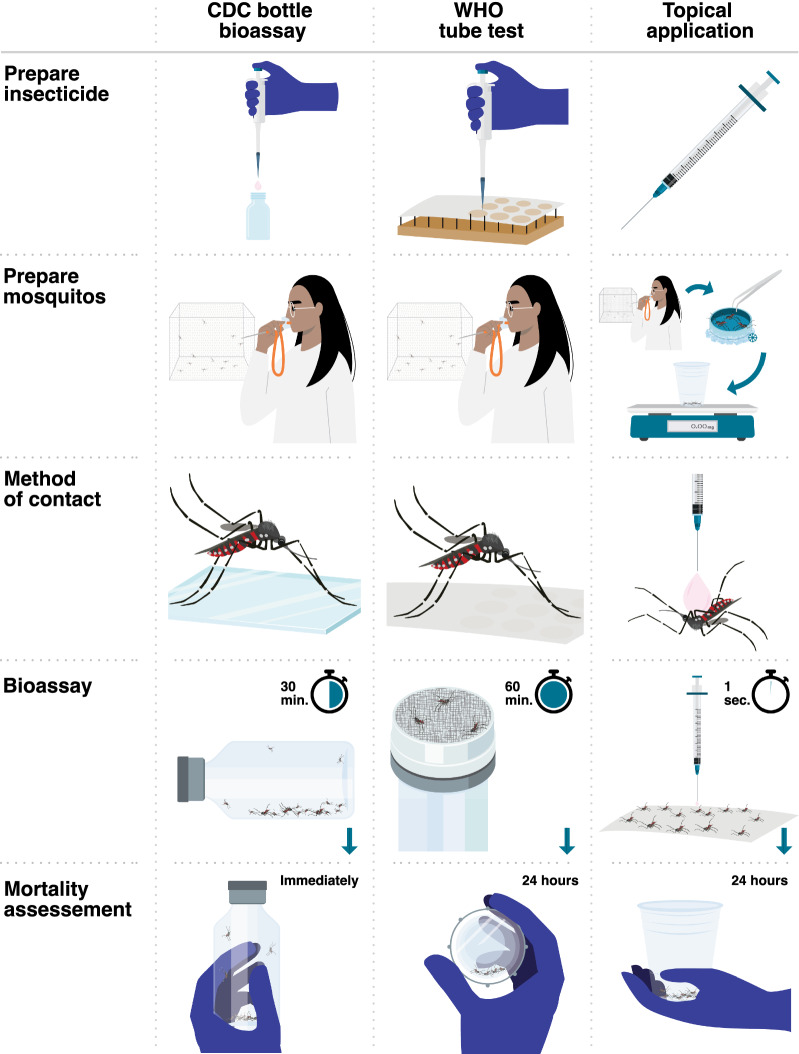
Overview of the differences between the CDC bottle bioassay (left column), WHO tube test (middle column), and topical application bioassay (right column). See methods section for details

There are important differences in how the different bioassays function and what they measure. First, the method of insecticide contact varies, with CDC bottle and WHO tube tests exposing mosquitoes via tarsal contact and topical application assays via direct application on the cuticle. Exposure time in the CDC bottle bioassay is 30 min, whereas it is 1 h in the WHO tube test, and in the topical application bioassay exposure is instantaneous due to direct application. In the WHO tube test, the insecticide is mixed with a carrier oil and impregnated on paper. Preparation of these papers is centralized, and papers can be ordered from Universiti Sains Malaysia. The concentrations that mosquitoes are exposed to in CDC bottle bioassay and topical assays are typically prepared by each research site, though pre-prepared concentrations for the CDC bottle bioassay can be requested from the CDC free of charge [20]. The WHO tube test provides an untreated resting place at each end of the cylinder tube where the mosquitoes could be unexposed to the insecticide for a period or the entire duration of the test. Mortality assessment in the CDC bottle bioassay occurs at the end of exposure time of 30 min, whereas it is 24 h after exposure in the WHO tube test and topical application. Handling of mosquitoes is different as well, with mosquitoes being knocked down by CO_2_ or ice in the topical application and handled using tweezers and brushes, whereas mosquitoes are transferred exclusively via manual aspiration in the other two assays. Lastly, the topical application assay assesses the dose per milligram of mosquito by controlling for the average weight of each mosquito cohort, whereas weight is not controlled in the CDC bottle or WHO tube test (Fig. 1). These differences between assays could lead to differences in determination of the insecticide susceptibility of mosquito populations.

Several studies have been conducted to compare two of the most widely used bioassays, CDC bottle bioassays and WHO tube tests, which are highly conflicting. Some studies report poor agreement [21–23], some high levels of agreement [23–25], and some intermediate levels [23, 26–29], particularly when comparing moderately resistant populations. Few studies have compared the topical application bioassay with other existing bioassays for mosquitoes. One study in France on *Aedes aegypti* and *Aedes albopictus* used both topical application and WHO tube tests to establish dose–response curves and found fairly similar resistance ratio values, though it should be noted that the mosquitoes were highly susceptible, so resistance ratio values were low and thus potential differences difficult to detect [30]. However, another study in Florida, USA, found that the topical application bioassay was able to detect differences in insecticide susceptibility in field mosquitoes that were not detected using the CDC bottle bioassay [18], and a similar study in Malaysia showed that the WHO tube test was not able to identify field strains as resistant, while the topical application assays identified both as resistant [17]. In all the above studies, differences were observed in particular in moderately resistant populations, which is logical since variation can only be observed when mean mortality is below 100% and above 0%, which is where they fall. Worryingly, though, it is the moderately resistant populations that we should aim to identify early and accurately.

Quantitative dose–response assays of field populations will provide more accurate information on the insecticide susceptibility status of the mosquito populations than threshold assays [18, 23, 29, 31]. Indeed, intensity assays for CDC bottle bioassays [7, 32] and WHO tube test [5] are now recommended and used more widely, though the limited number of dosages tested will not be suitable to perform dose–response analyses [33]. A dose–response analysis requires a higher number of mosquitoes and, depending on the variability in the data, more replicates are needed for statistical power. With mosquito numbers already being a limitation in current field bioassays, this is one of the main hurdles in collecting this type of data. High levels of variability have been observed in CDC bottle bioassays and also to a certain extent in WHO tube tests [22, 29]. With the dose that mosquitoes pick up in topical bioassays being highly controlled, the deviation in mortality for topical application assays is expected to be lower. Here we compare the CDC bottle bioassay, the WHO tube test, and the topical application bioassay side-by-side using an *Ae. aegypti* strain that is resistant to pyrethroids. Our main objective was to compare variability within the assays when dose–response curves are established with an identical inbred mosquito strain, under controlled laboratory conditions performed by the same researcher.

## Methods

### Study design

#### Dose–response curves for each insecticide susceptibility bioassay

Four replicates of four to six different deltamethrin (DM) concentrations were performed to construct dose–response curves for three different bioassays: CDC bottle bioassay, WHO tube test, and topical application bioassay. These concentrations were chosen by performing preliminary range-finding bioassays that resulted in mortality ranging from 0 to 100%. All bioassays were performed between 8:30 am and 4:30 pm and at ambient conditions (21 ± 2 °C, 23 ± 3% relative humidity [RH]). In all bioassays, a mosquito was considered “dead” if unable to hold itself upright or fly in a coordinated motion. All CDC bottles, WHO tubes, and topical plastic cups were labeled with random IDs for blind mortality assessment and were performed by the same researcher. Individual dose replicates were performed on different days to avoid day-to-day bias.

#### Fixed-dose experiment

To further assess the variance between the insecticide assays, 10 replicates were performed at one fixed dose, close to the lethal concentration/dose that kills 50% of exposed mosquitoes (LC_50_ or LD_50_). If preliminary experiments revealed replicates with either 0% or 100% mortality, a higher or lower dose, respectively, was used to be able to assess the full range of variation around the mean. These assays were completed in two separate experiments for each assay, thus five replicates of the same dose on the same day, with the same group of mosquitoes, similar to during a typical WHO tube test and CDC bottle bioassay.

### Mosquito species and testing facility

The *Ae. aegypti* MC1 (Maricopa County) strain was used in this study. The eggs of this pyrethroid-resistant strain were collected in the Phoenix area of Maricopa County, Arizona, USA, in 2018, and continuously reared in an insectary since then. MC1 is a homozygous resistant strain and possesses two known point mutations in the *kdr* gene: V1016I and F1534C (unpublished data). The V1016I mutation confers resistance to both permethrin and deltamethrin insecticides [34]. The F1534C mutation confers resistance to permethrin and other type I pyrethroids, in addition to organochlorides [19, 34–36], but it likely does not confer resistance to type II pyrethroids unless combined with another *kdr* mutation [36]. Mosquitoes were reared in an Arthropod Containment Level 1 (ACL-1) insectary facility at Arizona State University (USA) under standard rearing conditions in incubators set to 27 °C, 80% RH, and a 12:12-h photoperiod [12]. All mosquitoes tested were 2–5 days old, female, and non-blood-fed.

### Insecticide preparation and bioassays

Deltamethrin (Pestanal^®^, Sigma-Aldrich) solutions were prepared using the gravimetric method (using the mass of insecticide and mass of acetone rather than volume of acetone) [12]. Solutions were prepared in 15-ml Falcon tubes with the lid wrapped in parafilm (to reduce evaporation). Tubes were covered in aluminum foil (to prevent UV exposure), placed in a resealable plastic bag (to reduce evaporation), and stored at −20 °C to further prevent evaporation. Solutions were allowed to sit at room temperature for at least 1 h until use.

#### CDC bottle bioassays

General procedures were followed as described in the CDC bottle bioassay guidelines [7]. To create dose–response curves, five glass Wheaton^®^ 250-ml bottles were individually coated with a different DM concentration and one bottle coated with only acetone for the control. To coat the bottles, 1 ml of insecticide solution (or acetone) was pipetted into the bottles. The bottles were capped and maneuvered so that insecticide covered all parts of the bottles and caps. The bottles were then uncapped and placed on a bottle rotator (Cole-Parmer^®^) for 15 min to allow the insecticide to evenly coat the bottles and the acetone to evaporate. The bottles were stored uncapped in the dark for a minimum of 1 h and a maximum of 23 h until use in the assay. Approximately 25 (95% CI: 22.7–28.3) mosquitoes were aspirated into the bottles, and the mosquitoes were exposed in the bottles for 30 min, after which knockdown (inability to stand on legs or have coordinated flight, i.e., “dead”) was assessed [37].

#### WHO tube tests

Procedures were followed as described in the standard operating procedure for testing insecticide susceptibility of adult mosquitoes in WHO tube tests [5]. To prepare the insecticide-treated papers, filter paper (Whatman™ No. 1) was cut into 12 × 15 cm dimensions. Deltamethrin concentrations were prepared by mixing the insecticide with acetone and olive oil (MP Biomedicals, Fisher Scientific). Olive oil was used instead of silicone oil as it is less viscous and led to more accurate concentrations. The DM solutions were pipetted drop-by-drop in a grid onto the individual papers. The control paper was treated with acetone and olive oil only. Papers were allowed to dry in a fume hood for 24 h and subsequently stored in a 4 °C fridge, individually wrapped in aluminum foil. When ready to use, each paper was placed into individual plastic exposure tubes from the WHO tube test kit (purchased from the Universiti Sains Malaysia, Vector Control Research Unit). Untreated filtered paper (cut in the same dimensions) was placed into individual holding tubes. Approximately 25 mosquitoes were aspirated into a holding tube. After 1 h, the mosquitoes were coaxed using tapping and a short burst of breath to move from the holding tube to the exposure tube lined with insecticidal paper for approximately 1 min until most mosquitoes had entered the exposure tube (mean number exposed was 24.0, 95% CI: 18.9–29.2). After 1 h of exposure, knockdown was recorded and mosquitoes were transferred back to the emptied holding tubes. They were provided with 10% sucrose solution and placed in an incubator at 27 °C and 80% RH. Mortality was recorded after 24 h. Papers were used up to six times, following WHO guidelines.

#### Topical application bioassays

Mosquitoes were aspirated out of a cage into falcon tubes, which were immediately capped and placed on ice. Mosquitoes remained on ice for at least 30 min before dosing occurred. After the mosquitoes were sufficiently knocked down, they were poured onto a tray in an ice box filled with ice, picked up using forceps, and placed into small plastic cups on ice, with each cup containing 25 mosquitoes. Each cup of mosquitoes was weighed to the nearest 0.1 g using a microbalance, and mosquitoes were thereafter dosed with 0.5 μl of control or insecticide solution using a precision glass syringe (Hamilton™ 80465, Fisher Scientific). After dosing, the mosquitoes were poured back into their respective plastic cups, provided with 10% sucrose solution, and placed in an incubator at 27 °C and 80% RH. Mortality was assessed 24 h later.

### Data analysis

Abbott’s correction was applied to control for natural mortality using mortality in the control bottle for each test that was run during the same period [38]. If mortality in the control group was above 20%, all concurrent tests were discarded and repeated. Two different dose–response analyses were performed to compare all three assays with both commonly used methods in the statistical program R v4.1.3 [39]. First, probit analysis was performed following the BioRssay script, similar to the available BioRssay package [40]. For this, Abbott’s corrected mortality was transformed into probit values and a generalized linear model (GLM) was fitted using log-transformed concentration (or dose) values with the quasi-binomial family to account for possible overdispersion. McFadden’s *R*-squared values for the fit were calculated as 1 − log likelihood_model_/log likelihood_null_. The LC_50_ for the CDC bottle bioassay and WHO tube test and the LD_50_ for the topical application bioassay were calculated with the corresponding 95% CI based on the standard error of the model. Next, *n*-parameter logistic regression was performed using the nplr package and the model with the optimal number of parameters based on a weighted goodness-of-fit estimator was chosen [41]. The LC_50_ and LD_50_ with their 95% CIs were estimated based on the standard errors of the optimal model. In the fixed dose experiment, the homogeneity of variance was assessed using Levene’s test, with subsequent pair-wise comparisons using the Bonferroni correction for multiple comparisons.

## Results

The mortality of the MC1 strain when exposed to the diagnostic dose for *Aedes aegypti* of the CDC bottle bioassay (10 mg/bottle) was 69.0%, and mortality with the diagnostic dose WHO tube test (0.03%) was 0% (established using only one replicate of 25 mosquitoes), both demonstrating that this strain is resistant against DM. Mortality in the untreated control bottles for the CDC bottle bioassays was 0% in all (6 out of 6 control bottles) dose-responses experiments, 4% in one of the WHO tube tests (1 out of 7 controls), and 8%, twice 4%, and twice 0% in the topical bioassays.

**Fig. 2:**
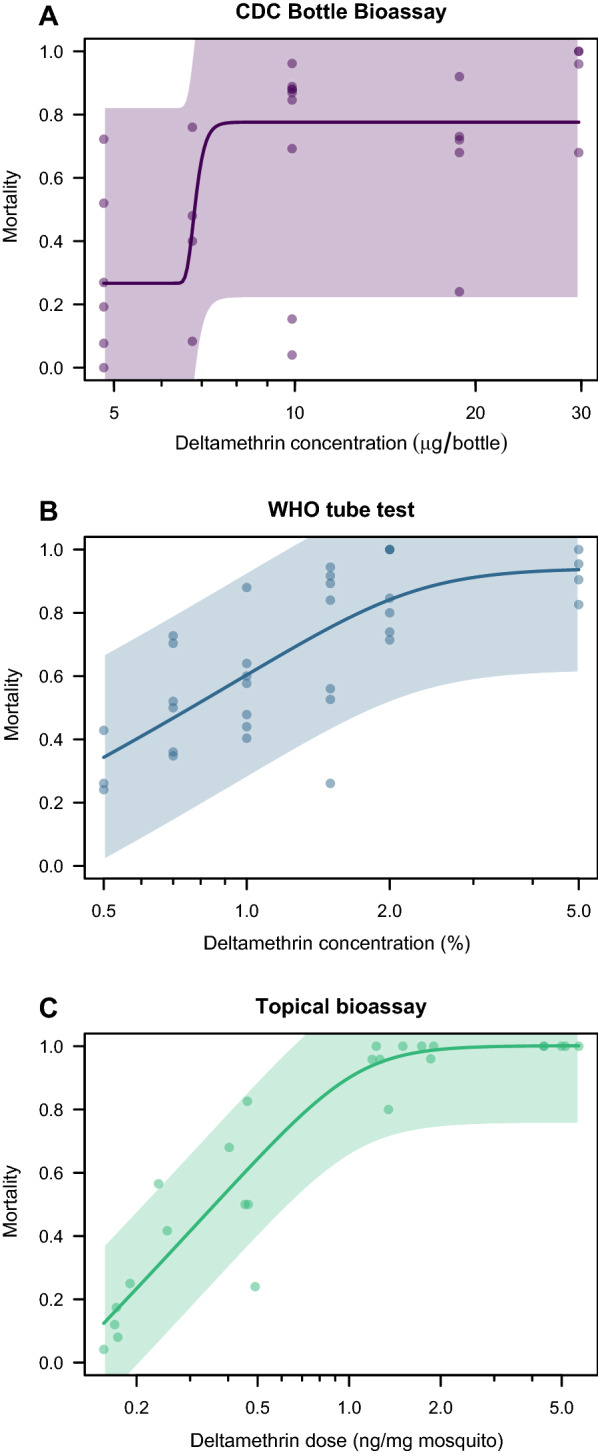
Dose–response data of deltamethrin-resistant *Ae. aegypti* female mosquitoes exposed to deltamethrin in CDC bottle bioassays (**A**), WHO tube tests (**B**), and topical application bioassays (**C**). Insecticide dose is presented on a log_10_ scale, mortality is presented on a normal scale. The trend line is based on five-parameter logistic regression with associated 95% confidence bands

In the n-parameter logistic regression, a five-parameter model had the highest goodness of fit for all three assays. The topical application bioassay had the highest goodness of fit (0.88), followed by the WHO tube test (0.55) and the lowest was the CDC bottle bioassay (0.31) (Fig. 2). The LC_50_ for the CDC bottle bioassay using the five-parameter logistic regression model was 6.81 µg/bottle (no reliable 95% CI could be estimated), the LC_50_ for WHO tube test was 0.76% (95% CI: 0.35–1.66) and the LD_50_ for the topical application bioassay was 0.36 ng/mg mosquito (95% CI: 0.23–0.59).

**Fig. 3:**
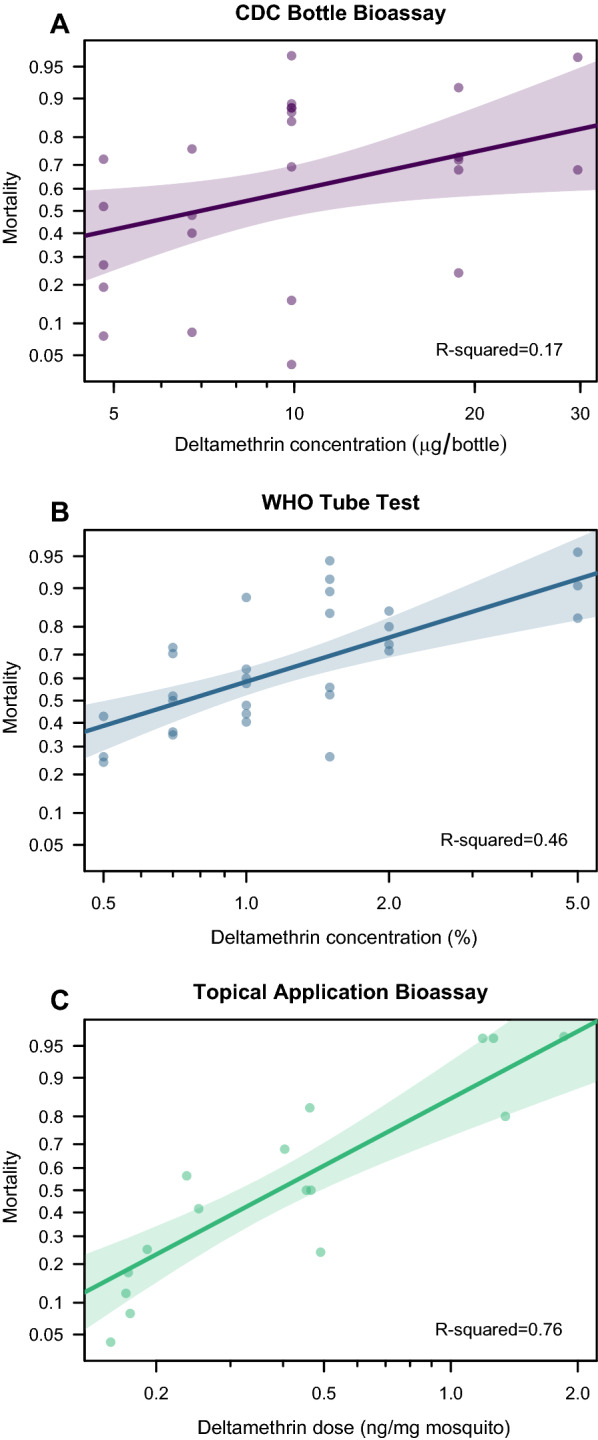
Dose–response data of deltamethrin-resistant *Ae. aegypti* female mosquitoes exposed to deltamethrin in CDC bottle bioassays (**A**), WHO tube tests (**B**), and topical application bioassays (**C**). Insecticide dose is presented on a log_10_ scale, mortality is presented on a probit scale. The trend line is based on general linear modeling with a probit link function, the McFadden *R*-squared value is given for each fit

For all three assays, insecticide concentration or dose was significantly correlated with mortality in the probit analysis (*P* = 0.038, *P* < 0.001, *P* < 0.001 for CDC bottle bioassay, WHO tube test and topical bioassay, respectively, Fig. 3). The variance in the dose–response curve was the lowest for the topical bioassay, with a McFadden *R*-squared value of 0.76, followed by the WHO tube test (*R*^2^_McFadden_ = 0.44), and the highest for the CDC bottle bioassays (*R*^2^_McFadden_ = 0.17). The LC_50_ calculation for the CDC bottle bioassay was 6.97 μg/bottle (95% CI: 4.0–12.1), the LC_50_ for the WHO tube test was 0.74% (95% CI: 0.56–0.99), and the LD_50_ for the topical bioassay was 0.39 ng/mg (95% CI: 0.31–0.49).

**Fig. 4:**
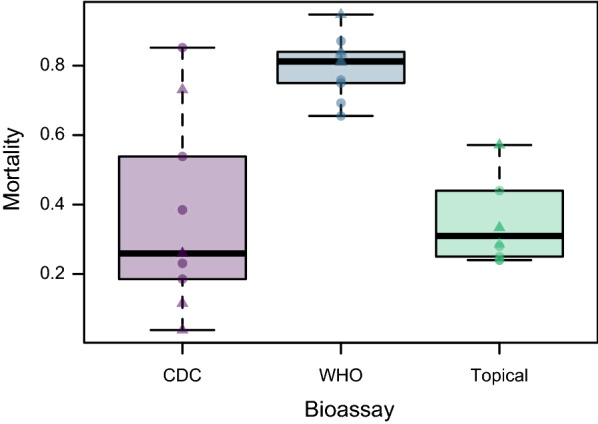
Variation assessment at a single dose for CDC bottle bioassay (3.89 ng/bottle), WHO tube test (1.0%), and topical application bioassay (average of 0.30 ng/mg mosquito). Circles and triangles denote data collected on two different days

To assess variation of the assays at one fixed dose, 10 replicates were exposed to 3.89 μg/bottle in the CDC bottle bioassay, 1% deltamethrin per paper in the WHO tube test, and a mean of 0.30 ng deltamethrin/mg mosquito (95% CI: 0.29–0.31, variation depending on mean mosquito weight per replicate). Mean mortality was 35.9% in the CDC bottle bioassay, 79.7% in the WHO tube test, and 35.5% in the topical application bioassay. All 10 replicates for each assay had mortality lower than 100% and higher than 0%; therefore, variance analysis could be reliably conducted (Additional files 1, 2, 3). A highly significant difference in the level of variance was observed between the different assays (Levene’s test, *F*_(2,27)_ = 6.3, *P* = 0.006, Fig. 4). Pair-wise comparisons showed a significantly higher variance in the CDC bottle assay than the WHO tube test (*P*  = 0.007; *P*_adj_ = 0.020), and higher variance in the CDC bottle assay than the topical bioassay, though this latter comparison was not significant following the Bonferroni correction (*P* = 0.043; *P*_adj_ = 0.13). No differences in variance were observed between the WHO tube test and topical application bioassay (*P* = 0.18; *P*_adj_ = 0.54).

## Discussion

The objective of this study was to compare the variation in mortality data from the CDC bottle bioassay, WHO tube test, and topical application bioassay. The most variation was seen within the dose–response curves generated by the CDC bottle bioassay, followed by the WHO tube test and finally the topical application bioassay (Figs. 2, 3). Less variation was observed when a single dose was tested with multiple replicates on a single day, demonstrating the added role of day-to-day variation. Yet, even with multiple replicates on a single day, considerably more variation was seen in mortality within the CDC bottle bioassay compared with the WHO tube test and topical application bioassay (Fig. 4). Increased levels of random variation in observed mortality would lead to a higher likelihood of error in determining the susceptibility status of mosquito populations, and thus impacting vector control decisions, as well as reduced power to establish whether susceptibly status is changing over time or is different between locations. To ensure sufficient statistical power to perform such comparisons, CDC bottle bioassays may need more replicate bottles than the WHO tube test and topical application bioassay. Currently, the CDC bottle bioassay recommends 80–100 mosquitoes in the form of four replicates of 20–25 mosquitoes per dose [7] and the WHO tube test recommends 100 exposed mosquitoes in the form of four replicates of 25 mosquitoes per dose [5], though the justification for these numbers is not given. No formal guidelines exist for the topical application bioassay, though if used to establish dose–response curves, the total number of mosquitoes used is larger than either CDC bottle bioassay or WHO tube test, as multiple doses need to be tested unless intensity assays are performed, which require two or three times the number of mosquitoes. If the topical application bioassay is used to determine resistance with a diagnostic dose [14], the optimal number of mosquitoes exposed will need to be formally established, similarly to how this was performed for bioassays with *Drosophila suzukii* [8].

There are a large variety of distinctions between the assays that could explain the difference in variation observed. First, the insecticide dose that mosquitoes pick up may not be the same for all mosquitoes within one replicate and between replicates in the WHO tube test and CDC bottle bioassay. Coating of insecticides in bottles might not be homogenous, even if care was taken by rotating bottles during the drying process with a bottle rotator. Caps, the neck, and the bottom of the bottle may have higher or lower concentrations, which means that mosquitoes could pick up different doses depending on where they land. In addition, the ability of the insecticide to bind to the glass is unknown, which could further lead to heterogeneous concentrations, particularly when the bottles are agitated, or tapped, when assessing mortality at the start of the experiment. As far as we are aware, there has been no research performed on the homogeneity of insecticide coating of bottles in mosquito bioassay or similar assays for other insects. The coating of insecticide on filter paper is suspected to be more homogeneous as it is performed on a flat surface, though due to the method of manual distribution of oil drops, it is still sensitive to heterogeneity in insecticide distribution. However, during the assay, mosquitoes have the ability to sit on the untreated surfaces on both ends of the tube, and thus the tube itself has a heterogeneous environment. A problem with tarsal contact assays is the crystallization of the insecticide, particularly on absorptive surfaces [42]. These crystals may result in an increase or decrease in toxicity, depending on their distribution and form [43, 44]. Crystallization is expected to be low on glass surfaces, but the process has been reported to be haphazard and difficult to predict for DDT formulations [43, 45]. Crystallization can be promoted by mechanical stimulation such as insect walking, surface scratches, or dust [42], though it is unclear whether movement by bottle rotation or tapping during mortality assessment at the start of the experiment would similarly lead to crystallization. Crystallization is a particular concern when insecticides are applied on absorptive surfaces, such as filter paper, which is why carrier oils are used to reduce crystallization. However, carrier oils themselves are also known to impact the availability and absorption of the insecticide for mosquitoes [42, 46]. Therefore, the insecticide dose used on a paper is manyfold higher than that used in the CDC bottle bioassay or topical bioassay for the same mortality. A second possible difference between these bioassays that could explain the observed variation is the different mortality time points and the associated bias in mortality counting. The CDC bottle bioassay assesses mortality at 30 min, versus mortality at 24 h in the WHO tube test and topical application bioassay. On the one hand, this leads to different phenotypes being assessed, as a knockdown phenotype at 30 min (which follows the mortality definition) may not lead to actual mortality at 24 h post-exposure and vice versa. This phenotype may inherently be more variable, depending on the complexity of the genetic pathway of resistance, as well as other genetic and environmental factors. Moreover, the assessment of mortality suffers from a large degree of subjectivity since a large variety of phenotypes may fit the definition of mortality with a significant gray area. Third, the variation seen within the CDC bottle bioassays and WHO tube tests could be due to a lack of controlling for mosquito weight. While weight differences were minor in these environmentally controlled inbred lab strains, the variation is expected to be much more pronounced in field populations, particularly when comparing populations from different seasons or ecological habitats [47–49]. A final important difference between these assays is the level of mosquito handling. While mosquitoes in the CDC bottle bioassay and WHO tube test are subjected to minimal handling using manual aspirators, mosquitoes in the topical application assay were anesthetized on ice before sorting and dosing using tweezers. This handling practice is expected to introduce some variation—especially since some mosquito groups are likely to spend more time on ice than others—although the observed variation in the topical application assay was the lowest of the three assays that were compared. Of note is that *Ae. aegypti* are particularly adaptable when exposed to cold [50]. It is not known to us whether such handling practices have a different impact on *Anopheles* mosquitoes, but CO_2_ can be used to anesthetize arthropods for topical application bioassays [12, 14].

Beyond the accuracy of each bioassay, there are other parameters important for the choice of optimal surveillance tool in each situation. First, there is the cost of the bioassay and the practicality of running these assays in remote field locations and the need to train staff. Overall, all three assays are low in cost, are portable, and can be performed in locations away from access to laboratory facilities, without extensive training. In addition, the most appropriate surveillance tool is largely determined by the objective of the study and the type of resistance present in the study area. Measuring 30-min knockdown in CDC bottle bioassays may overestimate mortality when metabolic resistance is abundant, since the detoxification of the insecticide may lead to later recovery of the mosquito [23]. If the objective of the study is to establish technical resistance, that is, to measure the resistance phenotype of a population under standardized conditions to compare how it changes over time or between different populations [10], then all three assays are appropriate, though our data suggest that the topical application assay may provide the highest accuracy, particularly when mosquito weight differs greatly across time and between different field sites. If the objective of the study is to establish whether a vector control tool is still effective in the face of insecticide resistance, then practical resistance assays should be developed that mimic natural exposure to insecticides of a field-relevant pool of mosquitoes under field-relevant environmental conditions. None of these bioassays is very suitable for this purpose (see [5, 10] for a discussion on this).

There are a few limitations to the present work. First, these results are based on a single mosquito species and strain, as well as a single insecticide. Further work will need to be performed to assess the generalizability of these findings. Specifically, the work performed here used *Ae. aegypti* as a model species, whereas CDC bottle bioassay and WHO tube tests are also frequently performed on *Anopheles* mosquitoes. However, there is little reason to assume that the level of variation presented here would differ for *Anopheles* mosquitoes. Different insecticides may also have different chemical structures that impact their binding to different materials impacting insecticide availability in the various assays. Whether this impacts random variation in the assays will need to be assessed. Next, a high level of variation was found in our study, particularly for the CDC bottle bioassay. Many studies do not report results of individual replicates, so it is difficult to assess whether this variation is out of the norm. However, some studies with these data included have also observed a high level of variation in bottle bioassay dose–response data [31, 51], though such high-level variation is not always seen [52]. Because individual dose replicates in the dose–response experiment in our study were performed on different days and assessed blindly, natural variation in day-to-day conditions of the mosquito pool, environment, and bottle coating were captured. In addition, since mortality assessments can be quite subjective after 30 min of exposure, blind assessment of different doses leads to the inclusion of this subjectivity in mortality assessment. In contrast, single dose assessment on a single day cannot be performed blindly and has an increased likelihood of bias towards lower variability. Indeed, in our second experiment with a single dose, lower variability was observed, which could be because of a lack of day-to-day variation, a reduction in unconscious bias in mortality assessment, or both. We expect the variation we measured in these experiments to be an underestimation of what would occur in the field where there would be additional lab-to-lab variation, researcher variation, mosquito genetic background variation, and environmental variation, all of which are likely to impact the phenotypic response to insecticides [10]. Indeed, when compiling dose–response data across different study sites, variation has been extensive [29, 53]. Finally, it is important to note that [54] the mosquitoes were exposed to controlled room temperature conditions during the handling and exposure (21 ± 2 °C, 23 ± 3% RH), which are both in terms of temperature and humidity lower than optimal conditions for these mosquitoes (rearing and post-exposure holding at 27 °C, 80% RH). While these conditions are likely to affect the mosquitoes and their susceptibility to insecticides [10], they are unlikely to explain the differences in variance observed between the different assays.

## Conclusion

Our data show that the topical bioassay generates the lowest amount of variation and thus is arguably the most accurate to establish technical resistance. In contrast, the CDC bottle bioassay led to high levels of random variation, and thus a low level of sensitivity in comparing populations over time or space, as seen previously [18]. These findings are crucial, especially since the CDC bottle bioassay is frequently used in the field, and its use may increase with newer insecticides and to determine resistance intensity. Our results imply that care should be taken in interpreting data coming from such assays, particularly when small samples have been used [8]. While both CDC bottle bioassays and WHO tube tests may be effective as an easy and crude method to diagnose highly resistant populations, topical application bioassays would perhaps be more suitable than current intensity tests for assessment and comparison of levels of resistance, particularly for low-level and moderate-resistance populations.

## Supplementary Information

**Additional file 1.** CDC Bottle Bioassay Data.

**Additional file 2.** WHO Tube Test Data.

**Additional file 3.** Topical Application Bioassay Data.

## Data Availability

The dataset supporting the conclusions of this article is included as Additional file: 1, 2 and 3.

## Abbreviations

95% CI: 95% Confidence interval
CDC: Centers for Disease Control and Prevention
DM: Deltamethrin
LC_50_: Lethal concentration that kills 50% of exposed mosquitoes
LD_50_: Lethal dose that kills 50% of exposed mosquitoes
MC1: *Aedes aegypti* strain isolated from Maricopa County
RH: Relative humidity
WHO: World Health Organization
